# Operational Differences between Product Development Partnership, Pharmaceutical Industry, and Investigator Initiated Clinical Trials

**DOI:** 10.3390/tropicalmed9030056

**Published:** 2024-02-29

**Authors:** Eric I Nebie, Peter van Eeuwijk, Hélène N. Sawadogo, Elisabeth Reus, Jürg Utzinger, Christian Burri

**Affiliations:** 1 Swiss Tropical and Public Health Institute, Kreuzstrasse 2, CH-4123 Allschwil, Switzerland; peter.vaneeuwijk@unibas.ch (P.v.E.); juerg.utzinger@swisstph.ch (J.U.); christian.burri@swisstph.ch (C.B.); 2 University of Basel, Petersplatz 1, CH-4003 Basel, Switzerland; 3 Centre de Recherche en Santé de Nouna, Street Namory Keita, Nouna P.O. Box 02, Burkina Faso; hsawadogo@crsn-nouna.bf; 4 Institute of Social Anthropology, University of Basel, Münsterplatz 19, CH-4051 Basel, Switzerland

**Keywords:** academia, clinical trial operations, efficiency, pharmaceutical industry, product development partnership

## Abstract

Medicine development is a lengthy endeavour. Increasing regulatory stringency and trial complexity might lead to reduced efficiency, dwindled output, and elevated costs. However, alternative models are possible. We compared the operational differences between pharmaceutical industry sponsored trials, product development partnership trials, and investigator-initiated trials to identify key drivers of inefficiency in clinical research. We conducted an exploratory mixed-methods study with stakeholders, including clinical trial sponsors, contract research organisations, and investigators. The qualitative component included 40 semi-structured interviews, document reviews of 12 studies and observations through work shadowing in research institutions in Burkina Faso, Mali, and Switzerland. The findings were triangulated with an online survey polling clinical research professionals. The operational differences were grouped under five categories: (i) trial start-up differences including governance and management structure; (ii) study complexity; (iii) site structural and organisational differences; (iv) study conduct, quality approaches, and standard operating procedures; and (v) site capacity strengthening and collaboration. Early involvement of sites in the planning and tailored quality approaches were considered critical for clinical operations performance. Differences between the types of trials reviewed pertained to planning, operational complexities, quality approaches, and support to the sites. Integration of quality-by-design components has the potential to alleviate unnecessary process burden.

## 1. Introduction

Clinical research has traditionally been, and continues to be, highly regulated and thus, structured in terms of procedures. Clinical operations refer to activities supporting clinical trials from early planning until completion. In addition to scientific inquiry, operations have a fundamental effect on, and mirror the success and efficiency of, product development. The ultimate goal of trial operations improvement is to complete trials with minimal essential resources and time while maintaining high quality. The main critical areas in clinical operations are planning, recruitment of participants, safety, and quality approaches [1,2]. This field is rapidly evolving; e.g., with the adoption of decentralised clinical trials and the revision of regulations. The COVID-19 pandemic highlighted the need for innovation and more flexibility in the clinical development of new medicines [3].

Operational efficiency requires multi-stakeholder approaches, including sponsors, funders, site managers, and contract research organisations (CROs) working at the interface of sponsors and sites [4]. Operations largely depend on the research settings (e.g., structures, infrastructure, and regulations), sponsors, and therapeutic fields. In the past two decades, there were several calls to action to address the organisational, operational, and regulatory pitfalls in clinical research. Various initiatives were launched, such as the Operational Efficiency Working Group (OEWG), aiming to address extensive timelines and processes, particularly in the field of oncology [4]. Other initiatives include the African Vaccine Regulatory Forum (AVAREF), a network of African national regulatory authorities and ethics committees aiming at harmonising processes across the continent, and the Swiss Clinical Trials Organization, which established a clinical trial complexity score supporting risk-based monitoring [5]. Different approaches were suggested to address the complexity in clinical trials [6,7] from design, process, management, and planning, and business model perspectives. Strategies for speeding up product development with parallel and overlapping clinical development phases have been implemented. However, these approaches tend to increase failure rates during the costly late phase III stage [8] and damper transparent decision-making in the early stage to abandon products with a high potential of failure. The development of adaptive clinical trial designs is an innovative approach to address the aforementioned issues [9]. It allows more flexibility and reduces delays between clinical trial phases. However, in some resource-constrained settings, its implementation may be challenging [10].

The suggested improvements could lead to a new era in clinical research and development. Yet, the clinical research ecosystem is perceived as ever bureaucratic and burdensome for investigators, sponsors, and study participants alike [11]. Clinical trial sponsors bear an important role and influence study operations at trial sites and in CROs that are often subcontracted by the sponsors.

Clinical product development is conducted through three main models: (i) pharmaceutical industry model (Pharma); (ii) product development partnership model (PDP); and (iii) academic model referred to as investigator initiated clinical trials (IITs). The latter is devoted mainly to resource-constrained settings for diseases that mainly affect disadvantaged and marginalised communities, such as malaria and neglected tropical diseases [12,13,14]. The increasing number of biotechnology companies share some of the lean approaches of the PDPs. Modern drug development often brings these players together for risk sharing. The sponsors differ in terms of business models, research and development focus, organisation, working culture, and governance structure, which might affect the outcomes of their clinical development activities.

IITs target critical medical needs, which are generally not in the primary focus for the industry [15]. In this model, investigators act as sponsors. IITs are mainly conducted by a single site or in a consortium approach and are led by clinical scientists [16]. Academia collaborates with Pharma and PDPs since it faces challenges to translate the rigorous good clinical practice (GCP) guidelines and address participant safety issues [17,18]. IITs are mainly funded by philanthropic organisations, governments, and public institutions (50%) through competitive grants [19]. In high-income countries, IITs are often conducted in academic teaching hospitals, whereas in low- and middle-income countries, IITs are generally implemented within research institutions addressing major public health issues. These institutions are often linked to disease control programmes, universities, and external partners [20]. Pharma and biopharmaceutical companies are for-profit organisations seeking to discover, develop, and deliver new products or optimise existing products. This business model focuses on market forecasts and returns on investments. This had led the interest of Pharma towards frequent non-communicable or severe rare diseases with expected high yields, leaving neglected (tropical) diseases, with poor market perspective, on the side [13,21]. However, this trend is changing in light of the movement for inclusion and diversity in clinical trials promoted by regulatory authorities, especially in the post- COVID-19 pandemic era. Finally, PDPs operate as non-profit think tanks committed to low-cost product delivery mainly for neglected (tropical) diseases. PDPs may focus on a “target disease” or a portfolio approach for innovation [22]. They coordinate funding, build efficient networks, and bring together a core team of drug development specialists with a wide array of insourced competence from academia, biotechnology companies, Pharma, dedicated services providers, and CROs [23,24] to accelerate product development [12,25]. In this model, the PDPs act as a virtual product developer [26] with low administrative costs [27].

We assessed the differences between drug development models and settings (high-income and resource-constrained settings) to identify the potential inefficiencies and corresponding lessons. This study focused on the planning, study complexity, team structures, quality approaches, and organisation from investigators, CROs, and sponsors’ perspectives.

## 2. Materials and Methods

### 2.1. Study Setting

This study was conducted in three well established research institutions; one based in a high income country (University Hospital Basel [USB], Switzerland) and two in resource-constrained settings (Unité de Recherche Clinique de Nanoro [URCN], Burkina Faso and Malaria Research and Training Centre [MRTC], Mali). All included research institutions have clinical research experience with Pharma sponsored trials and IITs. Additionally, the two African sites also host PDP sponsored trials. At USB, three clinics and the clinical trial unit that are all part of the Department of Clinical Research were included. At MRTC and URCN, the headquarters of the institutions and research sites where field and clinical research activities are being implemented were visited.

### 2.2. Study Design

We conducted an exploratory sequential mixed-methods study to assess the efficiency of drug development by comparing different models; namely, Pharma, IITs, and PDPs in terms of operations and organisations. The qualitative component was accomplished through work shadowing with direct observations and document reviews in the three research institutions, and semi-structured interviews with clinical trial CROs, investigators, and sponsors. The findings of the qualitative component informed an online survey conducted with clinical research investigators.

#### 2.2.1. Direct Observations

Direct observation consisted of a 6-week work shadowing in each research institution for a non-participatory observation of the operations, processes, and management of the site’s ongoing studies, using a predefined observation guide. We compared the organisation and processes of clinical trial implementation, including the participant recruitment process, workload by administrative activities, trial management, and quality management activities according to sponsor type in the three research institutions.

#### 2.2.2. Document Review

Two phase II or III ongoing or completed trials from two different sponsors (Pharma, IIT, and PDP) were selected in each site/unit for the document review. In total, 12 studies were reviewed (Table 1). The studies were selected from the clinical trials registration platform (clinicaltrials.gov) accessed on 24 August 2020 and cross-checked with the principal investigators and the site annual reports. The selection criteria included the trial phase (Phases II and III), type of sponsor, the period of study initiation (studies initiated from 1 August 2015 to 30 August 2020) and recruitment status. The documents assessed were the investigator site file and/or the trial master file (if applicable), ancillary trial documents (e.g., project management tools and field activities planning), and other general site management documentation. Paper versions of the available documentation on-site (protocol, forms, approvals, etc.) were directly assessed.

#### 2.2.3. Interviews

Semi-structured interviews were conducted with various clinical research stakeholders, including investigators and study coordinators, sponsors (project managers, project leaders, clinical development staff, quality responsibles, regulatory affairs responsibles, and medical officers), and CRO managers. The interviewees were selected based on experience in clinical research (i.e., at least 1 year of experience), current and past function in clinical trials. The Pharma sponsors and the CROs were purposively selected based on their clinical drug development experience and a diversified therapeutic portfolio in both high-income countries and resource-constrained settings. Interviewee profiles were described elsewhere [28]. Eight principal investigators had also experience acting as academic sponsors individually or on behalf of their institutions. The interviews were conducted in the interviewees’ preferred language (English or French). The main themes covered were related to participants’ experience with the different sponsors (previous and actual experience) in terms of study operations, study complexity including protocol complexity, quality approaches, and capacity strengthening and management differences between the sponsors.

#### 2.2.4. Online Survey

The online survey was developed on KoboCollect 1.30.1 (Kobo; Cambridge, MA, USA) and conducted with investigators with at least 1-year working experience in clinical trial operations in sub-Saharan Africa. The survey was grounded on the qualitative component results. The main themes covered included clinical trial processes and complexity, governance and management, and quality approaches. The survey was distributed to lists of clinical research professionals obtained from the WHO Special Programme for Research and Training in Tropical Diseases (TDR) clinical research career development programme and the European and Developing Countries Clinical Trials Partnership (EDCTP) that are leading organisations of research capacity strengthening in resource-constrained settings. Out of 190 potential participants invited for the survey, 80 responded (response rate 42%). Among the respondents, six were not eligible (four had less than 1 year of experience and two were not involved in trial operations).

### 2.3. Data Collection and Analysis

A scientific advisory board, consisting of four members, reviewed the tools before pilot testing. Guidelines were developed for the onsite observation and the document review. Specific interview guidelines were developed for each profile of the interviewee (i.e., sponsor, investigator, and CRO). The 6 week work shadowing took place from December 2020 to October 2021. Interviews were conducted face-to-face, on-site, or virtually using a Zoom license issues to the Swiss Tropical and Public Health Institute (Swiss TPH; Allschwil, Switzerland). The interviews were recorded, transcribed, translated, and coded. The coding and the analysis of the qualitative component were performed independently by two of the authors (E.I.N. and H.N.S.). A content analysis was performed for observations and document review data. The interview data were deductively analysed according to the predefined themes and categories in the interview guidelines using MAXQDA version 20 (VERBI Software 2021; Berlin, Germany) [28]. A descriptive analysis was performed for the online survey data using Stata Statistical Software: release 16 (StataCorp LLC; College Station, TX, USA). The study was reported following the mixed methods reporting guidelines [29].

### 2.4. Researchers Characteristics, Trustworthiness, and Reflexivity

There were no prior working experience of the research team with the visited sites. E.I.N. collected the data at all sites. The sites and trial sponsors were not involved in the design process, data collection, analysis, nor the funding of this study. Participation was voluntary and it was clearly mentioned to participants that the study was not commissioned by any clinical trial sponsor. Similarly, for the online survey, there was no power imbalance or hierarchical relationships between the providers of the contact lists (EDCTP and TDR) and the participants.

## 3. Results

Forty semi-structured interviews were conducted with sponsors, investigators, and members of CROs in both high-income countries and low- and middle-income countries. Participants had various working experiences, 70% with at least two sponsor types. All the visited sites/units had experience in conducting clinical trials as sponsor or sponsor-investigator. Four out of six participants from PDPs also had a Pharma background. Both interviewees and the online survey participants had expertise in various therapeutic areas, including infectious diseases and non-communicable diseases.

The online survey participants had diverse profiles and experiences, which are summarised in Table 2. Half of the participants had over a decade of experience in clinical research in various positions. Interviewees’ perceptions regarding drug development model operations and procedures were different according to their experience, the geographic location, and their working environment. The differences were grouped into five main categories: (i) trial start-up differences including governance and management structure; (ii) study complexity; (iii) sites structural and organisational differences; (iv) study conduct, quality approaches, and standard operating procedures (SOPs); and (v) contribution to site capacity strengthening and collaboration. The differences were found not only between the drug development models, but also within the same sponsor-type depending on the sponsor size.

### 3.1. Trial Start-Up Differences

#### 3.1.1. Sponsor’s Governance and Management Structure

The rigidity of the sponsor organisational structure with several layers of decisions making for study operational aspects was perceived as a major barrier while conducting clinical trials. On average, the layers of decision ranged from 1–2, 2–3, and 4–6, respectively in IITs, PDPs, and Pharma sponsored trials according to investigators in all the sites. The number of contact persons was different between the sponsors, too. In sub-Saharan Africa, the investigators stressed the high number of contacts as a driver of delays in conducting clinical trials, particularly in Pharma-sponsored trials, as mentioned by a senior scientist and principal investigator *“We know to whom we are talking when we work with PDPs”* (male, principal investigator, Burkina Faso). Or, as put by another interviewee: *“Large Pharma comes with a big structure and big teams and many layers of management and, you know, complications to the rollout of a trial and much more formalized processes”* (female, sponsor representative, Switzerland). Concerning communication, the following quote is of interest: *“In a PDP you’re much more able to pick up the telephone and ring your principal investigator and say, excuse me, doctor, please, can you contact your ethics committee? Because we’re expecting a response and it hasn’t come yet”* (female, sponsor representative, Switzerland).

**Figure 1:**
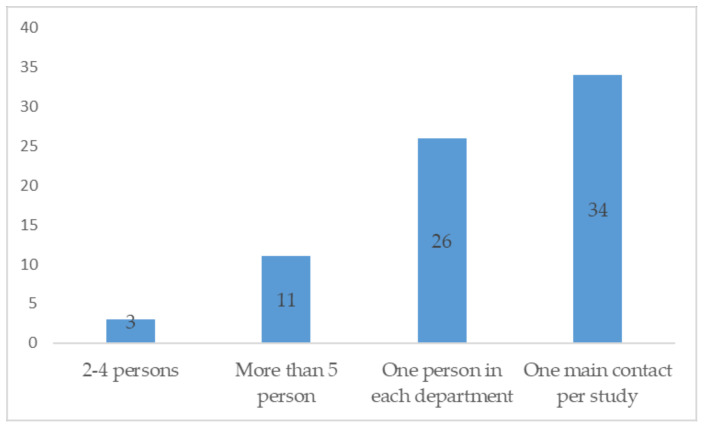
Investigators’ preferred number of sponsor contacts.

The investigators would prefer to have a single contact per department or one main contact for the study (Figure 1), as it is often the case in the PDP model or academia trials.

#### 3.1.2. Site Selection

For all sponsors, the main criteria for site selection were based on the principal investigator’s experience, previous working experience with the site, and the disease burden *“… having a strong principal investigator is key and a team below that”* (male, sponsor representative, Switzerland). Moreover, in the Pharma trials, the readiness of the site is also a key element of the site selection visit.

#### 3.1.3. Planning

Planning was a key element mentioned by most of the interviewees, while also stressing that some aspects, such as the regulatory application and ethical clearance timelines, are unpredictable, particularly in resource-constrained settings. The main differences in the planning phase were related to early involvement of sites in protocol development. In the PDP and IIT models with consortia, most of the investigators mentioned that they were more involved in the protocol development than in the industrial trials *“Generally, with PDPs you’re more involved in the genesis of the protocol, so interactions take place much further upstream than with Pharma companies where they have already the protocol”* (male, principal investigator, Mali). However, some principal investigators highlighted that they are currently witnessing a changing trend with most pharmaceutical companies, where upfront mechanisms are being established for the site’s contribution. From the online survey participants’ responses, there was no significant difference in terms of early involvement in trials according of the investigators experience in clinical trials (Table 3).

### 3.2. Study Complexity

#### 3.2.1. Study Design

Neither the investigators nor the sponsors perceived significant differences between sponsors in terms of study designs for the same therapeutic area and study phase, as mentioned by a principal investigator *“I don’t think there’s much difference, because the standards are practically the same, but the complexity will depend on the product being developed”* (male, principal investigator, Mali).

Only two out of 12 studies assessed pursued an adaptive study design and one study had a master protocol. The main study designs were parallel and factorial designs.

#### 3.2.2. Protocol Complexity

The interviewees perceived the protocol complexity to be similar when comparing PDP and Pharma sponsored trials. These trials are conducted with the same standards for a given phase of development and therapeutic area and target market authorization. When reviewing the protocols, the length of study protocols was also similar for the same therapeutic area and phase. The ancillary documents developed by the sites were consistent and similar across the assessed studies. However, in the online survey, 75% mentioned that there was a difference between sponsors with Pharma involved study protocols being more complex (Table 4).

### 3.3. Sites Structural and Organisational Differences

Structures of team organisations were different between high-income countries and resource-constrained settings, but similar within sub-Saharan African centres. The main actors on the sites are principal investigators, coordinators, clinical team leaders, laboratory team leaders, study physicians, laboratory technicians, nurses, and community field workers in Burkina Faso and Mali. In sub-Saharan Africa, the main activities are focused on study coordinators who are medical doctors, pharmacists, or biologists by training. On average, study coordinators had 8–10 years of experience in clinical research. The study coordinators often have additional responsibilities within the trials, combining sometimes study coordination with study lead physician or pharmacist or quality manager or even principal investigator roles. This was stressed by a junior study coordinator arguing, *“Very often, the study coordinator also acts as a lead physician”* (male, study coordinator, Burkina Faso). In Switzerland, the visited sites were hospital-based. The study coordinators were mainly pharmacists or nurses with an average of 2–3 years of experience in clinical research. The turnover of clinical research coordinators was quite high in the research units included in the current study in Switzerland.

The research team structure was the same regardless of the sponsor type in one of the centres. One of the senior scientists stressed the need to allocate the staff according to the trial needs. The study personnel allocation was independent from the type of sponsor. The main aspects considered were the trial phase and procedures. This was confirmed by the document review when reviewing the delegation logs. For instance, in two phase II studies (one PDP sponsored trial and one Pharma sponsored trial) in one of the visited sites, 26 staff members were involved in the conduct of each trial. The teams had the same structure and number of staff. The site organisation did not change according to the type of sponsor nor the size of the implementation team, but according to the expertise needed for the trial conduct. However, the investigators stressed that the procedures and the quality control were more stringent for the Pharma-initiated trials.

### 3.4. Study Conduct

#### 3.4.1. Differences in SOPs

A formal quality management unit/department was available in one of the sites, but all sites mentioned that there was a quality assurance mechanism in place at the institutional level. An investigator mentioned that the main additional SOPs requested by sponsors were related to the investigational product, which cannot be anticipated by the sites. The site procedures were compliant with most of the sponsor’s requirements.

The experiences of sponsors, investigators, and CROs were different regarding the difference in terms of study operations. CRO managers and investigators perceived the SOPs of PDPs as more adapted to the sites due to the focus on specific therapeutics as mentioned by a CRO manager *“So maybe their SOPs and the way they work is more adapted. Because they have a focus”* (male, CRO manager, USA).

**Figure 2:**
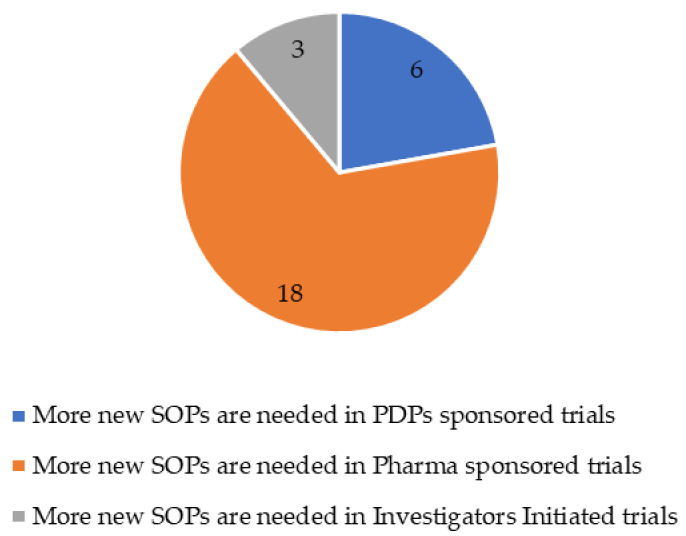
Need of new standard operating procedures by type of sponsor (N = 27).

Two thirds of the online survey participants who reported a difference between sponsors in terms of new SOPs needed (27 participants) stressed that these new SOPs were required when they work within Pharma trials compared to PDPs and academia (Figure 2).

#### 3.4.2. Quality of Clinical Trials

The perception of clinical trials quality was balanced among the investigators and CROs with more investigators considering Pharma sponsored clinical trials as more rigorous and of highest quality, particularly compared to IITs. However, they further mentioned that a continuous quality assurance could mitigate this difference: *“A caveat to be diligent enough is to assure quality and on the long run. So continuously assure quality. It is a dangerous or a compromise between the two models [Pharma and academia] that would likely be somewhat optimal”* (male, principal investigator, Switzerland). In sub-Saharan Africa, the PDPs and Pharma trials were considered similar by the interviewees: *“PDPs do practically what pharmaceutical companies do. It’s practically the same level of "standing" as with the pharmaceutical companies”* (male, principal investigator, Mali).

#### 3.4.3. Competitive Recruitment

The mode of allocation of recruitment targets differed between the different sponsors. Recruitment was mainly competitive in Pharma trials while in PDPs and IITs, the sites were allocated a sample of participants to be recruited according to the burden of disease. Competitive recruitment was perceived as an important hurdle in all the visited sites for different reasons. In Switzerland, the availability of various alternative treatments makes participant recruitment challenging, as mentioned by a principal investigator: *“So the golden years of clinical trials with industry [in Switzerland] are over. Global industry sometimes is astonished about pricing in such high-cost countries like Switzerland versus maybe Eastern countries. So it is a quite harsh pricing. Also in context of our main field (XX), plenty of drugs are approved leading to competency in trying to recruit patients that can also be treated with approved drugs. This is a struggle arising”* (male, principal investigator, Switzerland). For some diseases, the seasonality of transmission will also need further consideration when defining the recruitment target or selecting the sites.

#### 3.4.4. Logistics

Logistics were one of the main operational differences. In PDPs and IITs, except investigational products for non-licensed drugs, most of the procurements were performed in the countries where the trial was performed. However, in the Pharma model, most of the tools, equipment, and consumables to be used including case report forms and informed consent forms were shipped to the sites: *“A pharmaceutical company will send you, for example, case report forms that they have already made somewhere by plane or by boat, whereas we can do these things perfectly well! We even printed here, it is much cheaper. Otherwise, a PDP will probably let you print your CRFs here, as long as you agree on the content of the CRF, whereas a big company will want to do it and then transport it here, etc."* (male, principal investigator, Burkina Faso).

#### 3.4.5. Differences in Monitoring Approaches

Monitoring was assessed by comparing the duration, extent, number of visits, and level of support from CROs. There were differences in terms of CRO support to sites, with industry sponsor delegated CROs being more supportive to Pharma sponsored trials than IITs and PDPs. On a scale from 1 to 10, the median level of support was 8, 6, and 5 for Pharma, PDP, and IIT trials, respectively. Pharma trials also had more scheduled monitoring activities (number of visits and duration) compared to PDP and IIT trials. All the sites mentioned that risk-based monitoring was applied in their sites and they had internal monitoring teams who assess the trials before the formal sponsor monitoring. At USB, the clinical trial unit was in charge of monitoring most of the IITs conducted in the various departments.

### 3.5. Contribution to Site Capacity Strengthening

Sponsors’ contribution to site capacity strengthening is an important component to support studies operations. On the one hand, the investigators in low- and middle-income countries emphasised that institutional capacity strengthening is mainly driven by IIT and PDP sponsored studies, as mentioned by a principal investigator: *“PDPs are concerned with the development of our institutions, and I think they’re in a position to mobilise more resources than researchers, who, when looking for funding, are concerned about having a really fair budget to carry out their activities”* (male, principal investigator, Mali).

On the other hand, sponsors emphasised that capacity strengthening is not the primary focus of industry, although this is now changing for some settings, as mentioned by a project leader: *“Overall, I would say, knowing the Pharma industry quite well, that [capacity building] was not their primary focus”* (male, sponsor representative, South Africa). Or, put differently: *“So to give you an example, in [XX country], we built a YY bed ward with a bathroom and a monitoring unit like a conference room for the monitors. And so that value stays there when we are gone. And that means that more in my mind, that’s financially clever because you are enabling the institution”* (male, sponsor representative, South Africa).

### 3.6. Collaboration

Several investigators and sponsors mentioned the collaboration between sponsors and sites as a pillar of efficient study operations. Collaboration was not sponsor type specific as there were important differences driven by the level of trust and prior working experience with the site and support. However, PDPs were described as particularly close to the sites: *“We are closer to the field because we have more experience in the field. So we can be more pragmatic, we can make some changes, we cannot put things which make no sense, and that will not add anything to the project and maybe that will slow down the project. So, I think PDP members add something which is different and there is a collaboration which is very important for the success of the project”* (female, sponsor representative, Switzerland).

## 4. Discussion

The current study investigated efficiency in clinical research with a focus on study operations, comparing the three main drug development models—academia, Pharma, and PDPs. Investigators perceptions regarding IITs and Pharma trials were consistent across the sites in the three study countries Burkina Faso, Mali, and Switzerland. Investigators and CROs perceived substantial and heterogeneous differences between sponsors. These differences are not only related to the business model but also to the size of companies, their focus, and site locations. In light of the operational differences identified, we suggest potential strategies for improvement.

### 4.1. Planning: The Voice of the Site Investigators Is Critical

Investigators complained that they were not always involved early on in the design process, but they contributed to study participants’ recruitment targets for their site. In both, the university hospital and the research institutions participating in the current study, industry trials were found to be less inclusive and less flexible, thus, reducing innovation opportunities from the investigators’ perspectives. The lack of investigators involvement in the planning stage is a major pitfall delaying clinical trials. An early involvement of sites in trials planning could save time [2] and improve trials adaptability to sites. Despite the confidentiality agreements with sites, with the secrecy and the need of information protection, it urges sponsors to balance the level of information sharing and trust building during the conduct of a trial. The revision 2 of the International Conference of Harmonization (ICH) GCP put an emphasis on investigators empowerment. The involvement of researchers is also an opportunity for site’s capacity strengthening as junior or mid-career researchers will benefit from a positive exposure to upstream research processes. This could furthermore contribute to their professional career development. On another note, the guidance of investigators is critical to meet the countries regulatory and ethics committees’ requirements. Perhaps it could be more efficient that, when study sites are selected through calls for grant applications, the sites are offered the opportunity to design and adapt the study to their settings. This will improve the adoption and rollout of the product after its authorization for marketing in these settings.

The discontinuation of clinical trials during the COVID-19 pandemic, partly due to the logistical challenges for samples shipment [3], could have been mitigated through good planning and the use of local capacities. However, there was also a shift of market priorities with supply chain issues leading to the shortage of some reagents during the pandemic. For multicentre trials, there is need for standardisation of procedures across sites.

### 4.2. Improving Site and Team Organisation

The roles of different professionals in clinical trials are well codified. Although the investigator takes full responsibility of the trial conduct in the site, the roles of clinical research coordinators and trial managers are essential. The coordinator oversees all study operations [30]. Several training programmes have been developed to enhance clinical trial coordinators and managers skills. In our research, study coordinators turnover was higher in Switzerland compared to Burkina Faso and Mali. The same situation was reported for a shortage of clinical research professionals [31]. Metrics have been developed to assess the workload of clinical trial staff, particularly study coordinators, to maintain an efficient delivery of clinical trials [32]. The cumulative roles or navigation between several trials could be a strategy for staff retention. Caution is indicated for an accurate assessment of the workload and maintenance of optimal efficiency in clinical trials. In sub-Saharan Africa, for instance, study nurses have a somewhat limited role and responsibility, compared to what they could and often actually do in clinical research. Indeed, their role is often solely focused on the implementation [33]. Administrative support from nurses, given their in-depth site experience in clinical trials, was reported to contribute substantially to a reduction of the cost per participant recruitment on site by up to a quarter.

### 4.3. Strategies for Collaboration Improvement

An advantage of PDPs relies on the leaner communication flow with sites, compared to Pharma. For IITs, the management process is even much leaner. However, the reporting procedures of some funders of IITs are less flexible and extended. The collaboration with sites should go beyond single studies and be established as sustainable partnerships. Collaborations and partnerships based on trust [34] and transparency could contribute to the anticipation of trial implementation challenges, such as logistics and consideration of socio-cultural aspects. These collaborations should also include investigator benefits from the direct scientific outputs of the research, for instance, in terms of access to data for publication.

### 4.4. Towards More Equitable Site Selection

It is important to consider potential trial sites with less emphasis on the principal investigator and prior track records. Indeed, having a blinded selection with validated guidelines will avoid favouritism [35]. This also prevents perpetuation of the Matthew effect of well-established sites being preferably selected. Instead, participant recruitment capabilities should be prioritised [36]. Sponsor preference for established sites, while these centres are often overburdened and overwhelmed with the conduct of trials, jeopardizes the opportunity for diversification. Competing trials in some of the sites might even hamper the quality and the accrual capabilities of the sites. Offering the chance to explore new sites will provide more insights for timely recruitment and innovations. In resource-constrained settings, in fact, smaller sites and those located in rural settings were found to be more efficient in the recruitment of participants compared to larger sites with competing activities [37]. It is important to tailor the selection criteria based on disease burden and the real accrual capabilities of the sites.

### 4.5. Optimal Quality Approaches

The perceived differences between sponsors in terms of quality might rely on the workload caused by quality activities rather than the outputs of the study. The same staff are involved in all the studies and their experience is beneficial for all types of trials. By applying a one-size-fit-all approach to quality in sites, sites are overburdened with minor and non-critical findings resulting in unnecessary queries. Quality control was perceived as burdensome, particularly for industry-sponsored trials in terms of the frequency, extent, and management of the trials. The rationale of performing several monitoring visits in sites with the likelihood of critical deviation should be assessed. Hence, clear categorisation of monitoring visit findings would be ideal, using a risk-based approach. Planning visits according to study milestones could be an optimal approach. Site feasibility and initiation visits should be well performed in all trial sites. The quality-by-design concept promotes this approach. Of note, quality-by-design refers to a set of principles aiming to improve the design, conduct, and monitoring of clinical trials [38]. It is based on critical quality control factors [39]. The establishment of quality tolerance limits the need to be set for proper study management [40] and based on the trial phase and requirements, whilst site specificity can improve the quality approach, particularly in resource-constrained settings. While quality-by-design and innovative trial designs are meant to ensure the prompt conduct of clinical trials, external factors such as the regulatory and ethics applications could damper these efforts.

### 4.6. Capacity Strengthening

Capacity strengthening at clinical trial sites is an essential pillar in the PDP model for sustainable collaboration [41]. Capacity strengthening activities are better designed through investigator-initiated studies or partnerships through consortia driven by the investigators. Sustainable site capacity strengthening is necessary to maintain sites productivity. As such, site capacity strengthening should be based on the site’s needs and the institution’s vision. The pharmaceutical industry has a growing interest for capacity strengthening, mainly driven by the newly established global health departments and mandates through their corporate social responsibilities.

### 4.7. Strengths and Limitations

This study addressed the operational differences in clinical research by comparing investigator-initiated clinical trials, Pharma-sponsored trials, and PDPs. This study provided insights from various clinical research stakeholders’ perspectives and suggested solutions for operations improvement. Some of the findings are site specific and might not be generalizable. Indeed, there is considerable variability among sponsors.

Given the selection of studies included in the documents reviewed here that were readily obtained from the most widely used trial registration platform (i.e., Clinicaltrial.gov), might lead to a bias, as non-registered studies or trials registered in other platforms were missed. To mitigate this potential selection bias, we cross-checked the trial sites annual reports and discussed with principal investigators on the sites.

## 5. Conclusions

The evolution in the field of clinical research favoured the improvement of sites support from different types of sponsors. Despite the perceived similarities in study design and settings, there were multifactorial operational differences. The differences pertained to sponsors’ governance structure and management, capacity strengthening efforts, and planning. Early involvement of sites in trial planning and adoption of tailored and adapted quality approaches are critical for clinical operations improvement for all sponsors. The efficacy of a clinical trial is hampered by a complex management structure and the involvement of multiple contact layers on the sponsor side. For studies conducted in resource-constrained settings, operational adaptation to research ecosystems implies deep knowledge of the site and partnership that is built on trust. It is hoped that the revision of the clinical trials guidelines with a focus on quality-by-design, including critical-to-quality factors and quality tolerance limits, will further alleviate the unnecessary process burden in clinical research, contributing to fostering innovation in the design, conduct, and outcomes of clinical trials.

## Figures and Tables

**Table 1:** Number, phases, and status of reviewed studies in the three sites by sponsor-type.

Institution	Investigator Initiated Trial (IIT)			Product Development Partnership (PDP)			Pharmaceutical Industry Sponsored Trial (Pharma)		
	Number of Studies	Phase	Status	Number of Studies	Phase	Status	Number of Studies	Phase	Status
MRTC	1	IIIb-IV	C	1	III	C	1	II	O
URCN	1	IV	C	1	II	C	1	II	O
USB	3	II (2) I/II (1)	2 (O) 1 (C)	Not applicable	-	-	3	II (2) I (1) *	2 (C) 1 (O)
	5			2			5		

C = Completed, O = Ongoing; * Oncology trial.

**Table 2:** Demographics, eligibility, and experiences of online survey participants (N = 74).

Participants’ Characteristics	N (%)
Age in years	
20–40	34 (46.0)
41–50	30 (40.5)
>50	10 (13.5)
Sex	
Male	58 (78.4)
Female	16 (21.6)
Eligibility *	
Eligible	74 (91.8)
Non eligible	6 (8.2)
Number of years working in clinical trials	
1–5	17 (23.0)
6–10	24 (32.4)
11–15	20 (27.0)
>15	13 (17.6)
Experience with sponsors **	
Academia	57 (48.7)
Pharma	32 (27.4)
PDP	28 (23.9)

* One-year working experience in clinical trials operations. ** Some of the participants had experience with several types of sponsors.

**Table 3:** Online survey participants involvement in trial planning by sponsors according to investigator’s experience (N = 74).

Sponsor Type	Experience (in Years)				*p*-Value *
	1–5	6–10	11–15	>15	
Pharma sponsored trials	6 (18.8)	14 (43.8)	7 (21.9)	5 (15.6)	0.999
PDP sponsored trials	3 (10.7)	6 (21.4)	12 (42.9)	7 (25.0)	0.050
Academia sponsored trials	15 (26.3)	16 (28.1)	18 (31.6)	8 (14.0)	0.290

* Bonferroni adjusted *p*-value.

**Table 4:** Protocols complexity by sponsor-type according to online survey participants (N = 46).

Sponsor Type	N (%)
Academia	1 (2.2)
PDP	2 (4.4)
Pharma/PDP	17 (37.0)
Pharma	26 (56.5)
Total *	46 (100)

* Number of respondents among participants who have experience with at least two sponsor-types.

## Data Availability

The data are available on reasonable request.
